# Infant and young child feeding practices and associated socioeconomic and demographic factors among children aged 6–23 months in Ghana: Findings from Ghana Multiple Indicator Cluster Survey, 2017–2018

**DOI:** 10.1371/journal.pone.0286055

**Published:** 2023-06-09

**Authors:** Samson Akanbonga, Tanvir Hasan, Uzzal Chowdhury, Adrita Kaiser, Fatema Akter Bonny, Ignitius Ezekiel Lim, Ilias Mahmud

**Affiliations:** 1 Department of Nutrition and Dietherapy, Holy Family Hospital, Techiman, Ghana; 2 BRAC James P Grant School of Public Health, BRAC University, Dhaka, Bangladesh; 3 Save the Children International, Dhaka, Bangladesh; 4 Department of Comparative Biomedical Sciences, School of Veterinary Medicine, Louisiana State University, Baton Rouge, Louisiana, United States of America; 5 Department of Public Health, College of Public Health and Health Informatics, Qassim University, Al Bukairiyah, Saudi Arabia; TERI School of Advanced Studies, INDIA

## Abstract

**Background:**

Association between poor infant and young child feeding (IYCF) practices and malnutrition in infants and young children (IYC) is well established. Furthermore, appropriate IYCF practices are important during the first 1,000 days of life to ensure optimal health and development. Understanding IYCF practices and associated socioeconomic and demographic factors will inform interventions to achieve the UN 2030 Sustainable Development Goal (SDG) target to end malnutrition in all forms.

**Objective:**

This study estimates the prevalence of Minimum Dietary Diversity (MDD), Minimum Meal Frequency (MMF), and Minimum Acceptable Diet (MAD), and examines their association with socioeconomic and demographic characteristics among children aged 6–23 months in Ghana.

**Method:**

We used data from the Ghana Multiple Indicator Cluster Survey 6 (GMICS6) conducted in 2017–18. Participants were recruited through multi-stage stratified cluster sampling. Information on caregiver’s self-reported breastfeeding status and 24-hour dietary recall of foods IYC were fed with were collected through face-to-face interviews. We estimated the prevalence of MDD, MMF and MAD with a 95% confidence interval (CI). We investigated the socioeconomic and demographic determinants of MDD, MMF and MAD using univariate and multivariable logistic regression analyses.

**Findings:**

Among 2,585 IYC aged 6–23 months, MDD, MMF and MAD were estimated as 25.46%, 32.82% and 11.72% respectively. Age of the IYC, educational status of the mothers/primary caregivers, and resident regions were found to have positive associations with MDD, MMF and MAD. In addition, the richest household wealth index and urban area of residence were found to have significant positive associations with MDD.

**Conclusion:**

We report a low prevalence of MDD, MMF and MAD. Efforts to improve IYCF practices among children aged 6–23 months in Ghana should focus on multi-sectorial approaches including increasing access to formal education, income-generating activities and addressing regional and rural-urban inequity.

## Introduction

Appropriate feeding practices for infants and young children (IYC) reduce the risk of diarrhoea [1] and acute respiratory infections [2] and prevent anthropometric growth failure [3]. Appropriate feeding of IYC is therefore important particularly during the first 1,000 days of life to ensure optimal health and development [4]. The United Nations Convention on the Rights of the Child states that every child has the right to be raised well and get basic needs, which include a balanced diet, adequate clothing, sufficient shelter, and proper healthcare [5]. United Nations Children’s Fund (UNICEF) and the World Health Organisation (WHO) recommend initiation of breastfeeding within one hour of birth, exclusive breastfeeding for the first six months of life and introduction of nutritionally adequate, age-specific and safe complementary (solid, semi-solid or soft) foods at six months along with continued breastfeeding up to two years of age and beyond [6].

To assess infant and young child feeding (IYCF) practices, WHO and UNICEF (2021) developed 16 core indicators. These indicators include ever breastfed, early initiation of breastfeeding, exclusively breastfed for the first two days after birth, exclusive breastfeeding for the first six months, feeding formula and/or animal milk in addition to breast milk under six months, continued breastfeeding till 12–23 months, the introduction of solid, semi-solid or soft foods (ISSSF) at six months, minimum dietary diversity (MDD) during 6–23 months, minimum meal frequency (MMF) during 6–23 months, minimum milk feeding frequency (MMFF) for non-breastfed children aged 6–23 months, minimum acceptable diet (MAD) during 6–23 months, egg and/or flesh food consumption during 6–23 months, sweet beverage consumption during 6–23 months, unhealthy food consumption during 6–23 months, zero vegetable or fruit consumption during 6–23 months and bottle feeding during 0–23 months. MDD, MMF and MAD are among the important indicators recommended by WHO for assessing complementary feeding practices among IYC aged 6–23 months [7].

Despite the WHO/UNICEF recommendation and the critical benefits related to IYCF, most studies have recorded suboptimal rates of appropriate IYCF practices. Analysis of the UNICEF global database reports global rates of 52.2% and 29.4% for MMF and MDD respectively, among children aged 6–23 months [8]. Syntheses of national survey data from 80 low and middle-income countries showed that only 21.3%, 56.2% and 10.1% of these countries had a prevalence of more than 50% in MDD, MMF and MAD, respectively [9].

In sub-Saharan Africa, data from demographic and health surveys between 2010 and 2016 revealed that 41.9% of children aged 6–23 months met MMF, 21% met MDD and 9.8% met MAD [10]. A community-based survey in Iseyin, a rural community in Oyo state, Nigeria reported the prevalence of MDD at 17.7%, MMF at 46.9% and MAD at 14.9% [11]. A similar study in the rural Damot sore district, Southern Ethiopia, found the prevalence of MDD at 16.5%, MMF at 94.5% and MAD at 16.3% [12].

In Ghana, the prevalence of appropriate IYCF practices for IYC aged 6–23 months is far below the WHO recommendation. As of 2008 in Ghana, the prevalence of MAD for children aged 6–23 months was 29.9% [13], but this declined to 13% in 2014 [14]. In a study based on six public health facilities in Ghana’s Accra metropolitan Assembly, MAD was estimated at 32% [15]. A cross-sectional study in the Kpandai district of Ghana reported the prevalence of MAD among IYC aged 6–23 months at 8.5% [7]. A community-based study in Northern Ghana reported the prevalence of MDD at 35.5%, MMF at 57.3% and MAD at 25.5% among children aged 6–23 months [16]. In 2021, an analysis based on the Ghana Micronutrient Survey of IYC aged 6–23 months revealed that 42% met MDD, 38% met MMF and 14% met MAD [17]. Suboptimal IYCF practices have been found to be directly associated with childhood malnutrition [18, 19]. Underweight (weight-for-age Z-score <-2SD), wasting (weight-for-height Z-score <-2SD), stunting (height-for-age Z-score <-2SD), micronutrient deficiency and overweight (weight-for-age Z-score ˃2SD) account for more than half of all infant and child mortality in developing countries [20].

Governments and stakeholders need nationally representative accurate and timely evidence to understand the current situation of IYCF practices for IYC aged 6–23 months and its associated factors. Knowing the factors influencing IYCF practices will help shape the development and implementation of evidence-based and effective interventions. In this context, our study aimed to provide information about the prevalence of MDD, MMF and MAD among IYC aged 6–23 months and their association with socioeconomic and demographic characteristics of the IYC in Ghana using nationally representative data from the most recent Ghana Multiple Indicator Cluster Survey 6 (GMICS6), conducted in 2017–18. The findings of this study are expected to assist key policymakers and programme implementers to identify areas requiring further improvements and design need-based and evidence-driven interventions. This will help improve appropriate IYCF practices for IYC aged 6–23 months in Ghana and in the long run contribute towards the target of ending malnutrition in all forms under the UN 2030 second Sustainable Development Goal (SDG) of zero hunger.

## Materials and methods

### Study design

A secondary data analysis of the Ghana Multiple Indicator Cluster Survey 6 (GMICS6) was done to estimate the prevalence of MDD, MMF and MAD and investigate its associated socioeconomic and demographic factors. The GMICS6 was carried out from October 2017 to January 2018 by the Ghana Statistical Service (GSS) in partnership with the Ministry of Health, Ministry of Education, Ministry of Sanitation and Water Resources, Ministry of Gender, Children and Social Protection, Ghana Health Service and the Ghana Education Service [21] with technical support from UNICEF [21].

The global MICS programme is an international multi-purpose household survey initiated by UNICEF in the mid-1990s with the goal of assisting countries to collect internationally comparable data on varied characteristics of women and children such as breastfeeding status and dietary practices. This data is used for evidence-based national development planning and initiatives and for monitoring progress made towards national goals and global commitments such as the SDGs [22].

### Study setting and participants

Ghana is a middle-income West African country which currently has 16 administrative regions. However, at the time GMICS6 was conducted, there were 10 administrative regions namely: Western, Central, Greater Accra, Volta, Eastern, Ashanti, Brong Ahafo, Northern, Upper East and Upper West [21].

This study utilised data from the mother/caregiver-child pair about breastfeeding and dietary practices of 2,585 IYC aged 6–23 months.

### Sampling

The GMICS6 employed a multi-stage stratified cluster sampling approach using a sampling frame based on the 2010 Population and Housing Census (PHC) [21]. GMICS6 selected census enumeration areas (primary sampling units) from both urban and rural areas in each of the then 10 regions of Ghana by using systematic probability sampling proportional to size. Further details of the sampling are reported in the 2017–18 MICS report [21].

The overall sample size of the GMICS6 was 13,202 households with a response rate of 99.4% [21]. All children under 5 years of age living in the sampled households were included in the survey. A total of 8,903 children under five years of age were listed in these households with a response rate of 99.7%. Out of these children, 2,585 were in the age range of 6–23 months and were included in this study.

**Fig 1:**
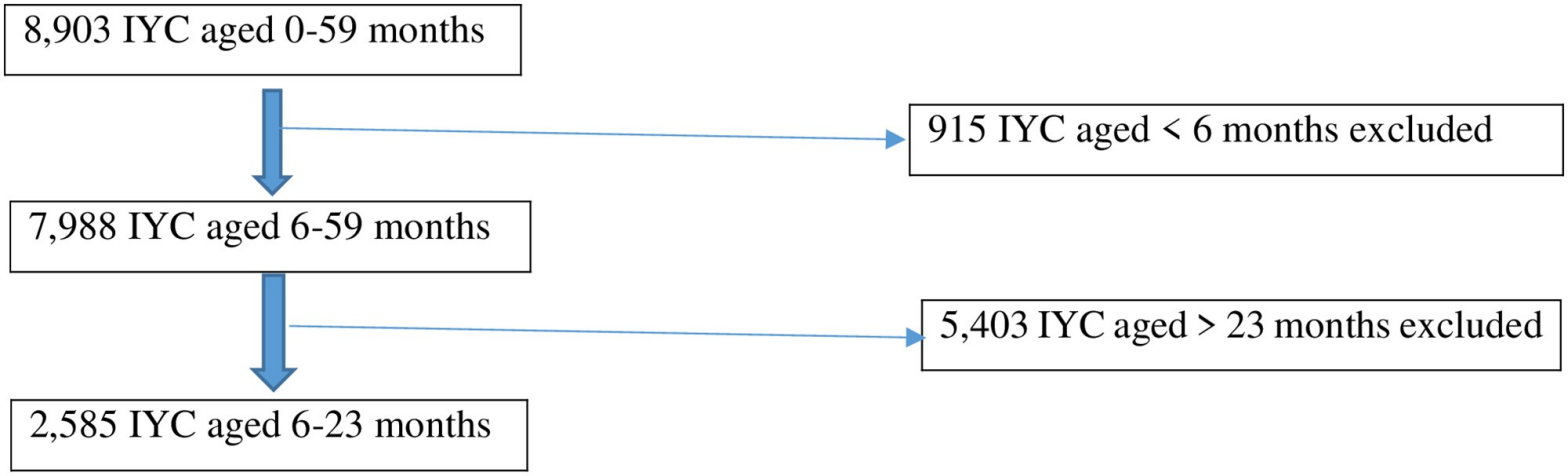
Sample flow details.

The sample flow details are outlined in Fig 1.

### Survey instruments

Mother’s or caregiver’s self-reported breastfeeding status and all foods that children under five years of age were fed with and frequency of feeding within the last 24 hours before the survey alongside socioeconomic and demographic characteristics were collected using the under-5 questionnaire of the GMICS6.

### Data collection

Data for the GMICS6 was collected via face-to-face interviews using Computer-Assisted Personal Interviewing (CAPI) technique.

A training of 30 days for field investigators was conducted from 10th September to 10th October 2017. The content of this training included interviewing techniques, contents of the questionnaires, CAPI application, skills in asking questions and mock interviews, 5 days of field practice and a one-day full pilot survey in localities around Winneba in the Central Region. Mandatory re-interviewing was implemented in one household per cluster using both random and purposive sampling techniques. Daily observations of the interviewer’s skills and performance were done [21].

### Ethical consideration

We utilised secondary data from GMICS6 whose ethical considerations have been published elsewhere [21].

### Measures/variables

#### Outcome variables

Minimum Dietary Diversity (MDD): IYC aged 6–23 months who were fed foods and beverages from at least five of eight food groups during the previous day. The eight food groups are: breastmilk; dairy products (milk, infant formula, yogurt and cheese); grains, roots, tubers and plantain; legumes or pulses (beans, peas and lentils), nuts and seeds; flesh foods (meat, fish, poultry and liver/organ meats); eggs; vitamin-A rich fruits and vegetables and other fruits and vegetables [23].

Minimum Meal Frequency (MMF): IYC aged 6–23 months who were fed foods and beverages 2 times and 3 times for 6–8 months and 9–23 months, respectively, and for non-breastfed IYC aged 6–23 months, 4 times including milk feeds and at least one solid, semi-solid or soft food during the previous day [23].

Minimum Milk Feeding Frequency (MMFF) for non-breastfed children: Non-breastfed IYC aged 6–23 months who were fed with at least two milk feeds (infant formula, animal milk or yogurt) during the previous day [23].

Minimum Acceptable Diet (MAD): Breastfed IYC aged 6–23 months who received MDD and MMF per age during the previous day and non-breastfed children aged 6–23 months who received MDD, MMF per age and MMFF during the previous day [23].

#### Independent variables

The following socioeconomic and demographic characteristics of IYC aged 6–23 months were included in the analysis: sex of the child; place of residence; administrative region (Western, Central, Greater Accra, Volta, Eastern, Ashanti, Brong Ahafo, Northern, Upper East and Upper West); age in months (6–8, 9–11, 12–17 and 18–23); mother’s or caretaker’s educational attainment (None, primary, JSS/JHS/middle, SSS/SHS/Secondary and higher); household ethnicity (Akan, Ga/Dangme, Ewe, Guan, Gruma, Mole Dagbani, Grusi, Mande and Others) and household wealth index which captures the underlying long-term wealth of the household ranked according to the wealth score of the household based on urban and rural factor scores regressed on assets owned by that household and finally divided into 5 equal parts or quintiles (poorest, poorer, middle, richer and richest).

### Statistical analyses

The GMICS6 dataset was accessed at mics.unicef.org. Each IYCF indicator or outcome variable (MDD, MMF and MAD) was dichotomised into a binary variable as “yes = 1” for a child who meets the indicator and “no = 0” for a child who does not meet the indicator. Frequencies, percentages/prevalences and means (± standard deviation) were used to describe the socioeconomic and demographic characteristics and outcome variables. Firstly, the Chi-square test was done to investigate the association between socioeconomic and demographic variables as well as the outcome variables. Socioeconomic and demographic variables that showed statistically significant (p ≤ 0.05) association with the outcome variables were also investigated using univariate (unadjusted) logistic regression. Statistically significant (p ≤ 0.05) socioeconomic and demographic variables in the univariate models were then included in the multivariable logistic regression model to get the adjusted association of selected socioeconomic and demographic factors after controlling for other potential confounders. The regression outputs were reported in the form of Odds Ratios (ORs) with 95% confidence intervals (CIs). The level of significance was determined at p-value ≤ 0.05. The data were analysed using Stata/SE version 17.0 (Stata Corporation, LLC, College Station, Texas, USA). Stata syntax developed for calculating IYCF indicators [23] was customised and used for the analysis. The Stata command ‘svyset’ was used to declare the complex sampling design of the data, while all estimations were performed by using the complex survey-specific command ‘svy.’

## Results

### Socioeconomic and demographic characteristics of infants and young children aged 6–23 months

Table 1 shows the socioeconomic and demographic characteristics of the IYC. The mean age of the sampled IYC was 14.3 months (±5.3) and over half (50.6%) were males. Among the IYC, 33.9% and 32.1% of them were 18–23 months and 12–17 months respectively while 56.6% were residing in rural areas. The Ashanti region recorded the highest percentage of IYC (23.9%) while the Upper West region recorded the lowest percentage of IYC (2.3%). In terms of education, about one-fifth (22.9%) of mothers or caregivers of the IYC had no formal education and only 5.9% had above secondary-level education. Majority of the IYC were from the Akan ethnic group (46.4%) while the Mande ethnic group recorded the least IYC (0.1%). Over a fifth (21.8%) of the IYC were from the poorest households and 19.8% were from the richest households.

**Table 1:** Socioeconomic and demographic characteristics of infants and young children aged 6–23 months (n = 2,585).

Socioeconomic and demographic characteristics	Frequency	Percentage (%)
**Sex**		
Male	1,309	50.62
Female	1,276	49.38
**Age (months)**		
6–8	501	19.37
9–11	377	14.58
12–17	830	32.11
18–23	877	33.94
**Area of residence**		
Urban	1,121	43.37
Rural	1,464	56.63
**Administrative region**		
Western	293	11.34
Central	248	9.58
Greater Accra	242	9.37
Volta	212	8.21
Eastern	297	11.47
Ashanti	620	23.99
Brong Ahafo	238	9.20
Northern	293	11.34
Upper East	83	3.20
Upper West	59	2.30
**Mother’s/caretaker’s educational level**		
None	577	22.92
Primary	554	21.62
JSS/JHS/Middle	999	37.13
SSS/SHS/Secondary	315	12.43
Higher	140	5.90
**Household ethnicity**		
Akan	1,201	46.44
Ga/Dangme	180	6.94
Ewe	283	10.93
Guan	110	4.29
Gruma	108	4.18
Mole Dagbani	441	17.06
Grusi	46	1.81
Mande	3	0.10
Others	213	8.25
**Household wealth index quintile**		
Poorest	564	21.84
Poorer	503	19.46
Middle	481	18.59
Richer	523	20.22
Richest	514	19.89

### Prevalence of minimum dietary diversity among infants and young children aged 6–23 months in Ghana

**Fig 2:**
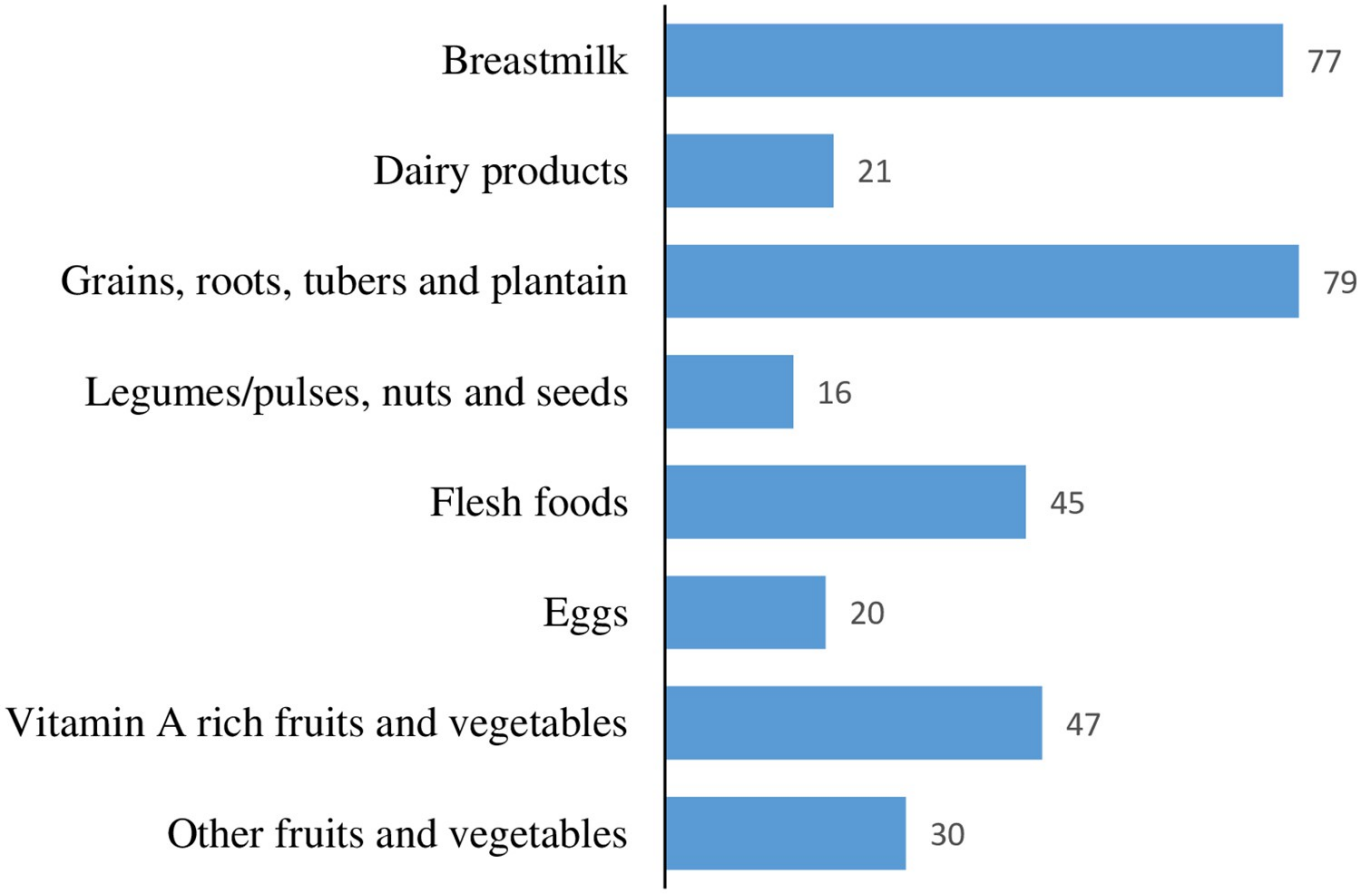
Percent of IYC fed with various food groups during the previous day.

Over a quarter (25.4%, 95% CI: 22.98, 27.94) of IYC in this study met MDD (Table 2). Almost equal proportions of male and female IYC achieved MDD, 24.7% and 26.2% respectively (Table 3). The majority (33.6%) of the IYC who achieved MDD were in the 12–17 months age group. Almost one-third of the IYC (31.7%) from urban areas achieved MDD whilst a fifth (20.6%) IYC from rural areas also achieved MDD. The Central region of Ghana recorded the highest prevalence of IYC who achieved MDD (34.7%) whilst the Upper West region recorded the least prevalence of MDD (13.9%). Nearly a fifth (18.4%) of IYC whose mothers/caregivers had no formal education achieved MDD whilst over half (52.4%) of IYC whose mothers/caregivers had above secondary-level education achieved MDD. The majority (94.6%) of IYC from the Mande ethnic group did not achieve MDD. In terms of household wealth quintile, nearly a fifth (18.4%) of IYC from the poorest households achieved MDD compared to almost half (46.7%) of IYC in the richest households who achieved MDD. The staples (grains, roots, tubers and plantain) were the food group that most (79%) IYC were fed with during the previous day (Fig 2). The majority of IYC (77%) were also fed breastmilk during the previous 24 hours. Consumption of other food groups by IYC was all below 50% with legumes or pulses, nuts and seeds recording a consumption of 16%.

**Table 2:** Prevalence of minimum dietary diversity, minimum meal frequency, minimum milk feeding frequency and minimum acceptable diet among infants and young children aged 6–23 months (n = 2,585).

IYCF indicator	Frequency	Prevalence (%)	95% CI
Minimum Dietary Diversity	658	25.46	22.98, 27.94
Minimum Meal Frequency	848	32.82	29.99, 35.66
Minimum Milk Feeding Frequency	88	15.91	11.89, 19.93
Minimum Acceptable Diet	303	11.72	9.72, 13.72

**Table 3:** Socioeconomic and demographic predictors of minimum dietary diversity among infants and young children aged 6–23 months.

Socioeconomic and demographic characteristics	Achieved MDD (%)	Chi-squared test (p-value)	Crude Odds Ratio (95% CI)	Adjusted Odds Ratio (95% CI)
**Sex**		0.6107		
Male	24.74			
Female	26.20			
**Age (months)**		≤0.001		
6–8	10.24		1.00	1.00
9–11	22.55		2.55 (1.46, 4.45)**	2.39 (1.32, 4.33)**
12–17	33.60		4.44 (2.67, 7.33)***	4.48 (2.69, 7.46)***
18–23	27.69		3.36 (2.14, 5.27)***	3.54 (2.18, 5.75)***
**Area of residence**		≤0.001		
Urban	31.71		1.00	1.00
Rural	20.67		0.56 (0.43, 0.73)***	0.85 (0.63, 1.14)
**Administrative region**		≤0.001		
Western	29.34		1.00	1.00
Central	34.74		1.28 (0.81, 2.02)	1.41 (0.90, 2.23)
Greater Accra	31.83		1.12 (0.67, 1.88)	0.64 (0.38, 1.06)
Volta	14.32		0.40 (0.20, 0.80)**	0.51 (0.26, 0.97)*
Eastern	20.80		0.63 (0.40, 0.99)*	0.71 (0.45, 1.11)
Ashanti	29.92		1.03 (0.66, 1.60)	0.91 (0.59, 1.40)
Brong Ahafo	18.52		0.55 (0.33, 0.91)*	0.56 (0.32, 0.97)*
Northern	21.47		0.66 (0.42, 1.02)	0.92 (0.56, 1.51)*
Upper East	19.50		0.58 (0.36, 0.93)*	0.74 (0.45, 1.22)
Upper West	13.90		0.39 (0.23, 0.65)***	0.49 (0.28, 0.84)*
**Mother’s/caregiver’s educational level**		≤0.001		
None	18.41		1.00	1.00
Primary	18.24		0.99 (0.61, 1.59)	0.85 (0.54, 1.33)
JSS/JHS/Middle	24.64		1.45 (1.03, 2.05)*	1.19 (0.78, 1.80)
SSS/SHS/Secondary	41.69		3.17 (2.07, 4.86)**	2.21 (1.34, 3.65)**
Higher	52.48		4.90 (2.44, 9.83)***	2.19 (1.11, 4.33)*
**Household ethnicity**		0.7894		
Akan	26.49			
Ga/Dangme	24.66			
Ewe	22.80			
Guan	19.40			
Gruma	20.45			
Mole Dagbani	27.35			
Grusi	18.75			
Mande	5.33			
Others	27.48			
**Household wealth index quintile**		≤0.001		
Poorest	18.49		1.00	1.00
Poorer	18.47		1.00 (0.67, 1.48)	0.86 (0.56, 1.33)
Middle	23.89		1.38 (0.93, 2.05)	1.13 (0.73, 1.74)
Richer	20.16		1.11 (0.71, 1.76)	0.76 (0.45, 1.30)
Richest	46.79		3.88 (2.58, 5.82)***	2.28 (1.33, 3.90)**

*p-value≤0.05,

**p-value≤0.01 and

***p-value≤0.001

### Prevalence of minimum meal frequency among infants and young children aged 6–23 months in Ghana

The percentage of IYC who achieved MMF in the 24 hours preceding the survey was found to be 32.8% (95% CI: 29.99, 35.66) (Table 2). Approximately, 3 in every 10 male IYC achieved MMF (31.3%) and 34.3% of female IYC had MMF (Table 4). IYC aged 6–8 months achieved the highest prevalence of MMF (53.7%) while 4 in every 5 IYC (80.2%) in the age group of 18–23 months did not achieve MMF. There was not much difference in the prevalence of MMF among IYC from urban and rural areas (32.1% and 33.7% respectively). The highest prevalence of MMF was recorded in the Central region (44.5%) while the Brong Ahafo region recorded the lowest prevalence (21.8%). Among the ethnic groups, the highest prevalence of MMF (35.5%) was observed among Mole Dagbani IYC. Contrastingly, none of the IYC from the Mande ethnic group achieved MMF. The prevalence of MMF increased with mother’s/caregiver’s educational status. Less than 30% (28.4%) and over half (55.6%) of IYC whose mothers or caregivers had no formal education and above secondary-level education achieved MMF respectively. The prevalence of MMF was higher among IYC from the richest households compared to those from the poorest households (39.5% versus 28.2% respectively).

**Table 4:** Socioeconomic and demographic predictors of minimum meal frequency among infants and young children aged 6–23 months.

Socioeconomic and demographic characteristics	Achieved MMF (%)	Chi-squared test (p-value)	Crude Odds Ratio (95% CI)	Adjusted Odds Ratio (95% CI)
**Sex**		0.3042		
Male	31.31			
Female	34.37			
**Age (months)**		≤0.001		
6–8	53.73		1.00	1.00
9–11	33.66		0.44 (0.31, 0.62)***	0.42 (0.29, 0.60)***
12–17	33.66		0.44 (0.31, 0.63)***	0.41 (0.29, 0.58)***
18–23	19.74		0.21 (0.15, 0.31)***	0.20 (0.13, 0.29)***
**Area of residence**		0.6014		
Urban	33.70			
Rural	32.15			
**Administrative region**		0.0013		
Western	31.17		1.00	1.00
Central	44.52		1.77 (1.14, 2.78)*	1.74 (1.12, 2.71)*
Greater Accra	24.54		0.72 (0.45, 1.15)	0.58 (0.36, 0.92)*
Volta	40.67		1.51 (0.95, 2.42)	1.58 (1.00, 2.50)
Eastern	27.49		0.84 (0.48, 1.45)	0.77 (0.42, 1.41)
Ashanti	37.89		1.35 (0.87, 2.07)	1.40 (0.92, 2.12)
Brong Ahafo	21.82		0.62 (0.36, 1.06)	0.61 (0.35, 1.04)
Northern	32.27		1.05 (0.68, 1.63)	1.18 (0.73, 1.93)
Upper East	25.48		0.75 (0.45, 1.26)	0.81 (0.47, 1.38)
Upper West	28.55		0.88 (0.58, 1.35)	0.86 (0.55, 1.36)
**Mother’s/caregive’s educational level**		≤0.001		
None	28.48		1.00	1.00
Primary	27.66		0.96 (0.65, 1.41)	0.94 (0.62, 1.40)
JSS/JHS/Middle	32.79		1.23 (0.92, 1.64)	1.20 (0.85, 1.69)
SSS/SHS/Secondary	39.83		1.66 (1.13, 2.45)*	1.92 (1.25, 2.95)*
Higher	55.66		3.15 (1.72, 5.79)***	3.23 (1.70, 6.14)***
**Household ethnicity**		0.1125		
Akan	34.60			
Ga/Dangme	27.10			
Ewe	34.66			
Guan	28.49			
Gruma	33.53			
Mole Dagbani	35.51			
Grusi	26.78			
Mande	0.00			
Others	22.89			
**Household wealth index quintile**		0.1076		
Poorest	28.04			
Poorer	33.27			
Middle	29.82			
Richer	33.68			
Richest	39.58			

*p-value≤0.05,

**p-value≤0.01 and

***p-value≤0.001

### Prevalence of minimum acceptable diet among infants and young children aged 6–23 months in Ghana

The prevalence of MAD 24 hours prior to the survey was 11.7% (95% CI: 9.7–13.7) (Table 2). Among males IYC, 10.6% achieved MAD while 12.8% of female IYC achieved MAD (Table 5). The highest prevalence of MAD was recorded among the age group of 12–17 months (15.1%) and only 7.6% of IYC in the age group of 6–8 months achieved MAD. About 13 in every 100 IYC (13.5%) from urban areas and about 10 in every 100 (10.3%) IYC from rural areas achieved MAD. In the Upper West region, 4.7% of IYC achieved MAD while 83.8% of IYC from the Ashanti region did not achieve MAD. Among IYC whose mothers or caregivers had no formal education, 8.2% achieved MAD and 33.6% of IYC whose mothers or caregivers had above secondary-level education achieved MAD. About 16 in every 100 IYC (16.8%) from the Mole Dagbani ethnic group achieved MAD while all IYC from the Mande ethnic group did not achieve MAD. The majority of IYC from the richest households achieved MAD compared to their poorest counterparts (18.5% and 9.9% respectively).

**Table 5:** Socioeconomic and demographic predictors of minimum acceptable diet among infants and young children aged 6–23 months.

Socioeconomic and demographic characteristics	Achieved MAD (%)	Chi-squared test (p-value)	Crude Odds Ratio (95% CI)	Adjusted Odds Ratio (95% CI)
**Sex**		0.2871		
Male	10.61			
Female	12.87			
**Age (months)**		0.0286		
6–8	7.60		1.00	1.00
9–11	13.49		1.90 (1.05, 3.44)*	1.86 (1.01, 3.41)*
12–17	15.17		2.17 (1.18, 3.40)*	2.13 (1.18, 3.83)*
18–23	10.06		1.36 (0.82, 2.26)	1.33 (0.77, 2.31)
**Area of residence**		0.1147		
Urban	13.55			
Rural	10.32			
**Administrative region**		≤0.001		
Western	11.59		1.00	1.00
Central	20.14		1.92 (1.10, 3.36)*	1.93 (1.09, 3.42)*
Greater Accra	7.17		0.59 (0.26, 1.35)	0.37 (0.16, 0.88)*
Volta	10.07		0.85 (0.40, 1.81)	1.00 (0.48, 2.05)
Eastern	6.92		0.57 (0.27, 1.20)	0.61 (0.27, 1.36)
Ashanti	16.14		1.47 (0.80, 2.70)	1.37 (0.74, 2.53)
Brong Ahafo	5.48		0.44 (0.21, 0.94)*	0.44 (0.20, 0.96)*
Northern	12.85		1.12 (0.58, 2.18)	1.50 (0.69, 3.30)
Upper East	7.61		0.63 (0.31, 1.27)	0.68 (0.32, 1.48)
Upper West	4.71		0.38 (0.17, 0.84)*	0.41 (0.17, 0.97)*
**Mother’s/caregiver’s educational level**		≤0.001		
None	8.23		1.00	1.00
Primary	7.48		0.90 (0.49, 1.64)	0.95 (0.51, 1.78)
JSS/JHS/Middle	10.67		1.33 (0.85, 2.08)	1.42 (0.83, 2.41)
SSS/SHS/Secondary	19.25		2.66 (1.51, 4.68)**	3.19 (1.66, 6.10)**
Higher	33.56		5.64 (2.20, 14.41)***	5.24 (2.04, 13.49)**
**Household ethnicity**		0.1662		
Akan	11.94			
Ga/Dangme	6.88			
Ewe	10.47			
Guan	10.88			
Gruma	6.81			
Mole Dagbani	16.86			
Grusi	5.80			
Mande	0.00			
Others	10.06			
**Household wealth index quintile**		0.0160		
Poorest	9.90		1.00	1.00
Poorer	7.96		0.79 (0.47, 1.31)	0.65 (0.38, 1.13)
Middle	12.15		1.26 (0.74, 2.15)	1.05 (0.57, 1.94)
Richer	10.25		1.04 (0.57, 1.90)	0.71 (0.34, 1.51)
Richest	18.50		2.06 (1.17, 3.63)*	1.01 (0.53, 1.93)

*p-value ≤0.05,

**p-value≤0.01 and

***p-value≤0.001

### Socioeconomic and demographic determinants of feeding practices for infant and young children aged 6–23 months

#### Minimum dietary diversity

Table 3 presents socioeconomic and demographic predictors of MDD among IYC aged 6–23 months. IYC from the age groups of 9–11 months (AOR: 2.39; 95% CI: 1.32, 4.33), 12–17 months (AOR: 4.48; 95% CI: 2.69, 7.46) and 18–23 months (AOR: 3.54; 95% CI: 2.18, 5.75) were 2.39, 4.48 and 3.54 times more likely to achieve MDD respectively compared to IYC aged 6–8 months. IYC having a mother or caregiver with secondary education (AOR: 2.21; 95% CI: 1.34, 3.65) and with above secondary education (AOR: 2.19; 95% CI: 1.11, 4.33) had 2 times the odds of achieving MDD compared to IYC with mothers or caregivers with no formal education. IYC from the richest households (AOR: 2.28; 95% CI: 1.33, 3.90) had over twice the odds of achieving MDD compared to IYC from the poorest households.

Compared to the Western region, IYC from the Volta (AOR: 0.51; 95% CI: 0.26, 0.97), Brong Ahafo (AOR: 0.56; 95% CI: 0.32, 0.97), Northern (AOR: 0.92; 95% CI: 0.56, 1.51) and Upper West (AOR: 0.49; 95% CI: 0.28, 0.84) regions respectively had 49%, 44%, 8% and 51% lower odds of achieving MDD. IYC from rural areas had 44% reduced odds of achieving MDD compared to IYC from urban areas.

#### Minimum meal frequency

Our multivariable logistic regression analysis suggests that IYC with mothers or caregivers having secondary education (AOR: 1.92; 95% CI: 1.25, 2.95) and above secondary education (AOR: 3.23; 95% CI: 1.70, 6.14) were 1.92 and 3.23 times more likely to achieve MMF respectively compared to the IYC with mothers or caregivers with no formal education. The odds of achieving MMF among IYC from the Central region (AOR: 1.74; 95% CI: 1.12, 2.71) was 1.74 times those from the Western region (Table 4).

On the other hand, IYC in the age groups of 9–11 months (AOR: 0.42; 95% CI: 0.29, 0.60), 12–17 months (AOR: 0.41; 95% CI: 0.29, 0.58) and 18–23 months (AOR: 0.20; 95% CI: 0.13, 0.29) had 58%, 59% and 80% reduced odds of achieving MMF respectively compared to IYC aged 6–8 months. IYC from the Greater Accra region (AOR: 0.58; 95% CI: 0.36, 0.92) had a 42% reduced odds of achieving MMF compared to IYC from the Western region.

#### Minimum acceptable diet

Our analyses suggest that IYC aged 9–11 months (AOR: 1.86; 95% CI: 1.01, 3.41) and 12–17 months (AOR: 2.13; 95% CI: 1.18, 3.83) were 1.86 and 2.13 times more likely to achieve MAD respectively compared to IYC in the age group of 6–8 months (Table 5). Having a mother or caregiver with secondary education (AOR: 3.19; 95% CI: 1.66, 6.10) and with above secondary education (AOR: 5.24; 95% CI: 2.04, 13.49) also put IYC at about 3 and 5 times the odds of achieving MAD compared to those with no education. IYC from the Central region had 1.93 times the odds of achieving MAD (AOR: 1.93; 95% CI: 1.09, 3.42) compared to those from the Western region.

On the other hand, IYC from the Greater Accra (AOR: 0.37; 95% CI: 0.16, 0.88), Brong Ahafo (AOR: 0.44; 95% CI: 0.20, 0.96) and Upper West (AOR: 0.41; 95% CI: 0.17, 0.97) regions had 63%, 56% and 59% reduced odds of achieving MAD respectively compared to IYC from the Western region.

## Discussion

We investigated the prevalence and socioeconomic and demographic determinants of MDD, MMF and MAD based on the GMICS 6 (2017/18). The prevalence of MDD, MMF and MAD was found to be 25.4%, 32.8% and 11.7% respectively. Socioeconomic and demographic characteristics having a positively significant association with MDD, MMF and MAD were the age of the IYC, educational status of the mothers/primary caregivers, and resident regions. In addition, the household wealth index was also found to have a positive significant association with MDD.

The percentage of IYC who achieved MDD in this study was slightly lower than 29.4% which was reported through an analysis of UNICEF global database [8] but comparable to the estimate of 25.1% based on the DHS data of 32 sub-Saharan African countries including Ghana from 2010 to 2020 [24]. The percentage of IYC who achieved MDD in the 24 hours preceding the survey in this study is higher than 21% found in a similar study using data from Ethiopia 2016 DHS [25] and 11.7% using data from Ethiopia 2019 DHS [26]. The percentage of IYC who achieved MDD in the 24 hours preceding the survey in this study is also higher than that reported in cross-sectional studies including 17.7% in Nigeria [11] and 16.5% in Ethiopia [12] but lower than those reported in Ghana including, 35.5% [16], 32% [15] and 34.8% [27]. The difference might be due to the difference in sample size and the season in which a particular survey was conducted.

Also, this study used the WHO and UNICEF revised definition of MDD (≥ five out of eight food groups) considering breastmilk as a distinct food group. Other previous studies however used the old definition (≥ four out of seven food groups). The use of the revised definition has been shown to result in a decrease in the prevalence of MDD [28]. This should be kept in mind in comparing the prevalence of MDD reported in this study with that of previous studies.

However, considering the results of this study, about a quarter of Ghanaian IYC aged 6–23 months were fed foods from at least five food groups during the previous day. This means about 75% of the IYC in this study are at risk of not meeting nutrient requirements which are essential to their physical and mental development. Even though quantities of the foods IYC were fed with were not included in this study and dietary intake was assessed on a one-day basis which may not reflect usual intake, nonetheless MDD has been shown to be a good predictor of nutrient adequacy and nutritional status [29].

This study also found a low intake of egg and flesh food among the IYC during the previous day (20% and 45% respectively). Many countries have also recorded similar low consumption of egg or flesh food [8]. This is worrisome given the fact that WHO recommends that IYC aged 6–23 months be fed with meat, poultry, fish or egg on a daily basis or as frequently as possible [23] to ensure optimal growth [30].

The percentage of IYC who achieved MMF in the 24 hours preceding the survey in this study is lower than 41.9% found in sub-Saharan Africa using DHS data [10] but similar to 33.6% found in a study in Nigeria [31]. Other community or facility-based studies in Ghana reported MMF at 39.5% [7], 46% [13], 38% [17], 58.2% [32] and 57.3% [16]. The difference might be attributed to the nationally representative nature of the data used in this study which allows for a more representative and heterogeneous sample and more reliable prevalence. The lower MMF in this study may be due to a lack of awareness among mothers or caregivers about the number of times IYC should be fed per day.

Ghanaian IYC aged 6–23 months in this study who achieved MAD compares favorably with the average of 9.8% found in sub-Saharan Africa using DHS data [10] and 9.2% in Southwestern Nigeria [31]. However, the percentage of IYC who achieved MAD in this study is also lower than that found in some community or facility-based studies including 27.8% [32], 25.5% [16] and 38.9% [33] in Northern Ghana, 33% in South Kivu, Democratic Republic of Congo [34], 14% in Southern Ghana [17] and 16.3% in Southern Ethiopia [12]. The reason may be that community or facility-based studies use a relatively more homogenous sample of participants compared to the data used in this study which is nationally representative. The low MAD (11.72%) in this study means that more than 80% of IYC were at increased risk of not meeting their micronutrient and meal frequency adequacy because MAD is a composite indicator of MDD and MMF which depicts both quality and quantity of food given to IYC. This low MAD may play a role in childhood malnutrition in Ghana. For instance, it has been observed that between 30% and 50% of all children aged 6–59 months in Ghana were malnourished [35]. This could negatively affect the physical growth and neurodevelopment of IYC in Ghana. This is because it has been noted that IYC who do not meet MDD, MMF or MAD are at higher risk of malnutrition [36, 37] which is significantly associated with neurodevelopment impairment [38].

The association of socioeconomic and demographic characteristics with MDD, MMF and MAD found in this study may help inform specific interventions to improve IYCF practices among children aged 6–23 months in Ghana. IYC in the age groups of 9–11, 12–17 and 18–23 months were 2.39, 4.48 and 3.54 times more likely to achieve MDD than those aged 6–8 months. Similarly, IYC aged 9–11 and 12–17 months were 1.86 and 2.13 times more likely to achieve MAD than those aged 6–8 months respectively. This is similar to that found in Dabat District, Ethiopia [39], districts of Bopa and Houeyogbe, Benin [40], India [41], Ghana [13], Southern Benin [42] and Northern Ghana [16]. The reason may be that younger infants and children are given only cereal-based porridge which does not contain diverse nutrients but older IYC are given family foods containing diverse nutrients making them more likely to meet MDD and MAD. Behaviour change communication targeting IYCF among children aged 6–23 months in Ghana should focus on increasing the production of locally available foods and how to modify various family foods in terms of consistency such as blending fruits and vegetables and how to enrich traditional porridges made from cereals with other foods such as groundnut, soybean, egg and milk to ensure diverse foods for younger IYC. Older IYC were also less likely to receive MMF than younger IYC (6–8 months). This means that attention is not given to IYC aged 9–23 months in terms of feeding frequency as compared to their younger counterparts. Sensitization is required for mothers/caretakers to make them understand that older IYC needs frequent meals to meet their nutrient requirements.

IYC of mothers/caregivers with secondary and/or higher education were more likely to achieve MDD, MMF and MAD than IYC of mothers/caregivers with no formal education. These results corroborate earlier studies such as that found in Ethiopia [26, 43–46], Ghana [47], India [41], South Kivu, Democratic Republic of Congo [34], in a systematic review [48] and Rwanda [49]. This may be due to the fact that educated mothers/caretakers can access information about IYCF through various mediums including the print medium. Educated mothers/caretakers also have a higher chance to be gainfully employed and can afford diverse foods for their IYC. Interventions to improve IYCF practices among children aged 6–23 months in Ghana should focus on improving access to formal education, especially for adolescent girls.

IYC from the richest households were 2.28 times more likely to achieve MDD than their counterparts from the poorest households. This finding is in agreement with that found in Pakistan [50], Ethiopia [44, 51, 52] and in Indonesia [53]. This may be due to the fact that the wealthiest households are able to afford diverse foods for their IYC as compared to the poorest households. Efforts to improve IYCF practices among children aged 6–23 months in Ghana should focus on providing access to income-generating activities to improve the economic conditions of households and hence improve MDD.

IYC from the Central region were more likely to achieve MMF and MAD than those from the Western region. Also, IYC from Volta, Brong Ahafo, Northern and Upper West regions were less likely to achieve MDD compared to IYC from the Western region, IYC from Greater Accra region were less likely to achieve MMF compared to those from Western region and those from Greater Accra, Brong Ahafo and Upper West regions were less likely to achieve MAD compared to those from the Western region. Region of residence as a significant factor to meeting IYCF practices has also been found in previous studies in Ghana [13, 47] and India [41]. This may be due to geographical differences and the kinds of foods produced in a particular region or other dynamics of a particular region.

Surprisingly, in this study, only MDD was significantly associated with the area of residence. The urban area of residence has however been shown to be associated with IYCF practices in previous studies in Ethiopia [25, 26, 39], and in South Kivu, Democratic Republic of Congo [34]. In this study, the lack of significant association between MMF and MAD with the area of residence may be due to the fact that the gap between rural and urban areas in Ghana in terms of feeding frequency is being narrowed while dietary diversity remains widely heterogeneous. Another possible explanation is that rural residents may be increasingly moving away from feeding their IYC with locally available diverse foods to commercially packaged foods. More efforts should be made to further limit the gap between rural and urban areas by improving road networks and other infrastructure.

### Strengths and limitations of the study

This study utilized the most recent nationally representative GMICS6 data with a large sample size (13,202 households with 2,585 IYC aged 6–23 months) and high response rate (99.4%). This study used the WHO and UNICEF definition of MMD which has been recommended for measuring intermediate outcome indicators for tracking progress towards the 2025 global nutrition targets [54]. The data used in this study has a limitation of having only one day of diet recall per child, which may not be representative of the day-to-day dietary intake and also prone to recall bias. In addition, the measure does not take into consideration the amount of food consumed and thus it does not completely measure nutrient adequacy. Nonetheless this method has been shown to be a good predictor of nutrient adequacy and nutritional status [29]. Lastly, the analysis was limited to variables in the dataset and only associations and no causality can be inferred due to the cross-sectional nature of the survey.

## Conclusion and recommendation

The prevalence of MDD, MMF and MAD among Ghanaian IYC aged 6–23 months in this study was low (25.4%, 32.8% and 11.7% respectively). Socioeconomic and demographic characteristics of IYC having positively significant associations with MDD were the age of IYC, educational status of mothers/caretakers, household wealth index, administrative region and area of residence. Additionally, the age of IYC, educational status of mothers/caretakers and administrative region were socioeconomic and demographic characteristics of IYC that showed significant positive associations with MMF and MAD. Surprisingly, unlike other previous studies, only MDD had a significant association with the area of residence in this study.

Based on the evidence generated in this study, we recommend that interventions towards MDD, MMF and MAD in Ghana should focus on improving access to formal education, income-generating activities and strengthening behaviour change communication using available channels in the communities to improve awareness and knowledge of IYCF.

## References

[1] Ogbo F. A., Ogeleka K. A. P., Woolfenden S., Page A., Eastwood J. and the Global Child Health Research interest group (2017). Infant feeding practices and diarrhoea in sub-Saharan African countries with high diarrhoea mortality. *PLos ONE*, 12(2):e0171792. doi: 10.1371/journal.pone.0171792 28192518 PMC5305225

[2] Ahmed K. Y., Page A., Arora A., Ogbo F. A. and the Global Maternal and Child Health Research collaboration (2020). Associations between infant and young child feeding practices and acute respiratory infection and diarrhoea in Ethiopia: A propensity score matching approach. *PLoS ONE*, 15(4): e0230978. doi: 10.1371/journal.pone.0230978 32236145 PMC7112197

[3] Perkins J. M., Jayatissa R. and Subramanian S. V. (2018). Dietary diversity and anthropometric status and failure among infants and young children in Sri Lanka. *Nutrition*, 55–56:76–83. doi: 10.1016/j.nut.2018.03.049 29980091

[4] Njeru, C. M., Ngugi, A., Kathomi, C. and Limbe, M. S. (2021). Complementary feeding: Is it the forgotten factor of the first 1000 days of life? Researchsquare.com. 10.21203/rs.3.rs-140037/v1. Accessed on December 26, 2021 @ 17:00 GMT.

[5] UNICEF (2019). The state of the world’s children, food and nutrition. Growing well in a changing world. UNICEF.

[6] WHO (2021). Infant and young child feeding. WHO. https://www.who.int/news-room/fact-sheets/detail/infant-and-young-child-feeding on 11/11/2021 at 12:24 GMT.

[7] Appiah K. B., Cheyuo E. K., Alhassan A., Ayanore M. A., Kubuga C. K. and Mogre V. (2020). Mothers’ knowledge and attitude regarding child feeding recommendations, complementary feeding practices and determinants of adequate diet. *BMC nutrition*, 6(67) no page number. 10.1186/s40795-020-00393-0 PMC7706028 33292706

[8] White J. M., Begin F., Kumapley R., Murray C. and Krasevec J. (2017). Complementary feeding practices: Current global and regional estimates. *Maternal and child nutrition*, 13(S2):e12505. doi: 10.1111/mcn.12505 29032623 PMC6865887

[9] Gatica-Dominguez G, Neves P. A. R., Barros A. J. D. and Victora C. G. (2021). Complementary Feeding Practices in 80 Low- and Middle-Income Countries: Prevalence of and Socioeconomic Inequalities in Dietary Diversity, Meal Frequency, and Dietary Adequacy *Journal of nutrition*, 151:1956–64. doi: 10.1093/jn/nxab088 33847352 PMC8245881

[10] Gebremedhin S. (2019). Core and optional infant and young child feeding indicators in sub-Saharan Africa: a cross-sectional study. *BMJ Open*, 9:e023238. doi: 10.1136/bmjopen-2018-023238 30782876 PMC6377519

[11] Ariyo O., Aderibigbe O. R., Ojo T. J. and Sturm B. (2021). Determinants of appropriate complementary feeding practices among women with children aged 6–23 months in Iseyin, Nigeria. *Scientific African*, 13:e00848.

[12] Areja A., Yohannes D. and Yohannis M. (2017). Determinants of appropriate complementary feeding practice among children 6–23 months of age in rural Damot sore district, Southern Ethiopia; a community based cross sectional study. *BMC Nutrition*, 3(82). 10.1186/s40795-017-0202-y PMC7050925 32153858

[13] Issaka A. I., Agho K. E., Burns P., Page A., and Dibley M. J. (2014). Determinants of inadequate complementary feeding practices among children 6–23 months in Ghana. *Public Health Nutrition*, 18(4):669–78. 24844532 10.1017/S1368980014000834 PMC10271301

[14] GSS/GHS/ICF International (2015). Ghana Demographic and Health Survey, 2014. GSS/GHS/ICF International.

[15] Gyampoh S., Otoo G. E. and Aryeetey R. N. O. (2014). Child feeding knowledge and practices among women participating in growth monitoring and promotion in Accra, Ghana. *BMC pregnancy and childbirth*, 14:180. doi: 10.1186/1471-2393-14-180 24886576 PMC4047542

[16] Saaka M., Larbi A., Mutaru S. and Hoeschle-Zeledon I. (2016). Magnitude and factors associated with appropriate complementary feeding among children 6–23 months in Northern Ghana. *BMC Nutrition*, 2(2).no page number.

[17] Donkor W. E. S., Adu-Afarwuah S., Wegmuller R., Bentil H., Petry N., Rohner F. et al. (2021). Complementary feeding indicators in relation to micronutrient status of Ghanaian children aged 6–23 months: Results from a national survey. *Life*, 11(969) no page number. doi: 10.3390/life11090969 34575118 PMC8468967

[18] Anees M., -ur-Rehman A., Ahmad A. M. R., Bukhari S., Siddique M. H., Farooq U., et al. (2020). Persistent inadequacies in infant and young Child feeding practices and their determinants. *Journal of Food and Nutrition Research*, 8(7):347–354.

[19] Marriott B. P., White A., Hadden L., Davies J. C. and Wallingford J. C. (2012). WHO infant and young child feeding indicators: associations with growth measures in 14 low-income countries. *Maternal and child nutrition*, 8(3):354–70. 22171937 10.1111/j.1740-8709.2011.00380.x PMC6860880

[20] Walson J. L. and Berkley J. A. (2018). The impact of malnutrition on childhood infections. *Current opinion in infectious diseases*, 31(3):231–6. doi: 10.1097/QCO.0000000000000448 29570495 PMC6037284

[21] GSS (2018). Multiple Indicator Cluster Survey (MICS 2017/18), Survey Findings Report. Accra, Ghana. GSS.

[22] Dey N. E. Y., Dziwornu E., Frimpong-Manso K., Duah H. O. and Agbadi P. (2020). Correlates of child functional difficulties status in Ghana: A further analysis of the 2017/18 multiple indicator cluster survey. *Heliyon*, 6:e05727. doi: 10.1016/j.heliyon.2020.e05727 33364496 PMC7750366

[23] WHO/UNICEF (2021). Indicators for assessing infant and young child feeding practices: definitions and measurement methods. WHO/UNICEF. http://creativecommons.org/licenses/by-nc-sa/3.0/igo. Accessed on November 13, 2021 at 19:27 GMT.

[24] Aboagye R. G., Seidu A.-A., Ahinkorah B. O., Arthur-Holmes F., Cadri A., Dadzie L. K., et al. (2021). Dietary diversity and undernutrition in children aged 6–23 months in sub-Saharan Africa. *Nutrients*, 13:3431. No page number. doi: 10.3390/nu13103431 34684435 PMC8537414

[25] Tassew A. A., Tekle D. Y., Belachew A. B. and Adhena B. M. (2019). Factors affecting feeding 6–23 months age children according to minimum acceptable diet in Ethiopia: A multilevel analysis of the Ethiopian Demographic Health Survey. PLoS ONE, 14(2):e0203098. doi: 10.1371/journal.pone.0203098 30789922 PMC6383941

[26] Shagaro S. S., Mulugeta B. T. and Kale T. D. (2018). Complementary feeding practices and associated factors among mothers of children aged 6–23 months in Ethiopia: Secondary data analysis of Ethiopian mini demographic and health survey 2019. *Archives of Public Health*, 79:205. doi: 10.1186/s13690-021-00725-x 34809724 PMC8607675

[27] Saaka M., Wemakor A., Abizari A. and Aryee P. (2015). How well do WHO complementary feeding indicators relate to nutritional status of children aged 6–23 months in rural Northern Ghana? *BMC Public Health*, 15:1157. doi: 10.1186/s12889-015-2494-7 26596246 PMC4656186

[28] Roy A., Hossain M., Khan S. A., Hanif A. A. M., Hasan M., Hossaine M., et al. (2021). Differences in estimates of minimum dietary diversity by using the new and old Definition: evidence from Bangladesh. *Current Developments in Nutrition*, 5(Supplement 2):808. doi: 10.1093/cdn/nzab046_105 PMC8992578 35415389

[29] Sealey-Potts C. and Potts Ac (2014). An association of dietary diversity and nutritional status of preschool children. *Austin journal of nutrition and food science*, 2(7):1040.

[30] Tang M. and Krebs N. F. (2014). High protein intake from meat as complementary food increases growth but not adiposity in breastfed infants: a randomized trial. *American Journal of Clinical Nutrition*, 100 (5):1322–28. doi: 10.3945/ajcn.114.088807 25332329 PMC4196483

[31] S. Folake O. and I. Ebunoluwa G. (2020). Complementary feeding practices and associated factors among nursing mothers in Southwestern Nigeria. *International journal of maternal and child health and AIDS*, 9(2):223–31. doi: 10.21106/ijma.363 32704409 PMC7370275

[32] Saaka M. and Abaah I. (2015). Maternal and infant factors associated with child growth in the first year of life. *Science*, 3(5):775–781.

[33] Anin S. K., Saaka M., Fischer F. and Kraemer A. (2020). Association between Infant and Young Child Feeding (IYCF) Indicators and the Nutritional Status of Children (6–23 Months) in Northern Ghana. *Nutrients*, 12:2565. doi: 10.3390/nu12092565 32847027 PMC7551146

[34] Kambale R. M., Ngaboyeka G. A., Kasengi J. B., Niyitegeka S., Cinkenye B. R., Baruti A., et al. (2021). Minimum acceptable diet among children aged 6–23 months in South Kivu, Democratic Republic of Congo: a community-based cross-sectional study. *BMC Pediatrics*, 21:239. 34011304 10.1186/s12887-021-02713-0 PMC8132412

[35] Kuwornu J., Amoyaw J., Manyanga T., Cooper E., Donkoh E. and Nkrumah A. (2020). Measuring the Overall Burden of Early Childhood Malnutrition in Ghana: A Comparison of Estimates from Multiple Data Sources. *International Journal of Health Policy and Management*, doi: 10.34172/ijhpm.2020.253 33589568 PMC9808187

[36] Andina E., Madinar M., and Achadi E. L. (2021). Fulfilment of Minimum Acceptable Diet as Dominant Factor in Wasting in Children Aged 6–23 Months in Central Jakarta, Indonesia, 2019. *Indonesia journal of public health nutrition*, 1(2). n.p.

[37] Benedict L., Hong S. A., Winichagoon P., Tejativaddhana P. and Kasemsup V. (2021). Double burden of malnutrition and its association with infant and young child feeding practices among children under five in Thailand. *Public health nutrition*, 24(10):3058–65. doi: 10.1017/S1368980020003304 33054885 PMC9884786

[38] Galler J. R., Bringas-Vega M. L., Tang Q., Rabinowitz A. G., Musa K. I., Chai W. J., et al. (2021). Neurodevelopmental effects of childhood malnutrition: A neuroimaging perspective. *NeuroImage*, 231(117828). doi: 10.1016/j.neuroimage.2021.117828 33549754

[39] Belew A. K., Ali B. M., Abebe Z. and Dachew B. A. (2017). Dietary diversity and meal frequency among infant and young children: a community based study. *Italian Journal of Pediatrics*, 43:73 doi: 10.1186/s13052-017-0384-6 28810887 PMC5558775

[40] Bodjrènon F. S. U., Hounkpatin W. A., Termote C. Dato G. and Savy M. (2020). Determining factors associated with breastfeeding and complementary feeding practices in rural Southern Benin. *Food Sci Nutr*., 9:135–144. doi: 10.1002/fsn3.1971 33473277 PMC7802539

[41] Dhami M. V., Ogbo F. A., Osuagwu U. L. and Agho K. E. (2019). Prevalence and factors associated with complementary feeding practices among children aged 6–23 months in India: a regional analysis. *BMC Public Health*,19:1034. doi: 10.1186/s12889-019-7360-6 31370827 PMC6676514

[42] Mitchodigni I. M., Hounkpatin W. A., Ntandou-Bouzitou G., Avohou H., Termote C, Kennedy Gina, et al. (2017). Complementary feeding practices: determinants of dietary diversity and meal frequency among children aged 6–23 months in Southern Benin. *Food Secience*, 9:1117–30. doi: 10.1007/s12571-017-0722-y

[43] Abebe H., Gashu M., Kebede A., Abata H., Yeshaneh A., Workye H. et al. (2021). Minimum acceptable diet and associated factors among children aged 6–23 months in Ethiopia. *Italian Journal of Pediatrics*, 47:215. doi: 10.1186/s13052-021-01169-3 34717712 PMC8557568

[44] Dagmawit S., Zewdie A. and Tegegne T. K. (2017). Minimum dietary diversity and associated factors among children aged 6–23 months in Addis Ababa, Ethiopia. *International Journal for Equity in Health*,16:181. doi: 10.1186/s12939-017-0680-1 29025434 PMC5639776

[45] Molla A., Egata G., Getacher L., Kebede B., Sayih A., Arega M. et al. (2021). Minimum acceptable diet and associated factors among infants and young children aged 6–23 months in Amhara region, Central Ethiopia: community-based cross-sectional study. *BMJ Open*, 11:e044282. doi: 10.1136/bmjopen-2020-044284 33972337 PMC8112428

[46] Mulaw G. F., Feleke F. W. and Masresha S. A. (2020). Maternal characteristics are associated with child dietary diversity score in Golina district, Northeast Ethiopia: a community-based cross-sectional study. *Journal of nutrition and metabolism*, Article ID 6702036. 10.1155/2020/6702036 PMC7528112 33029394

[47] Anane I., Nie F. and Huang J. (2021). Socioeconomic and geographic pattern of food consumption and dietary diversity among children aged 6–23 months old in Ghana. *Nutrients*, 13(2):603. doi: 10.3390/nu13020603 33673212 PMC7918505

[48] Scaglioni S., De Cosmi V., Ciappolino V., Parazzini F., Brambilla P. and Agostoni C. (2018). Factors Influencing Children’s Eating Behaviours. *Nutrients*, 10(706). doi: 10.3390/nu10060706 29857549 PMC6024598

[49] Van Der, S. (2015). The Association Between Complementary Feeding Indicators and Linear Child Growth and the Determinants of Inappropriate Feeding Practices Among Children 6–23 Months in Rwanda. Master’s Thesis, Wageningen University, Division of Epidemiology and Public Health, Wageningen, The Netherlands.

[50] Ali M., Arif M. and Shah A. A. (2021). Complementary feeding practices and associated factors among children aged 6–23 months in Pakistan. *PLOS ONE*, 16(2):e0247602. Nutrients, 13(603). doi: 10.1371/journal.pone.0247602 33630931 PMC7906416

[51] Tuji T. S. and Wake A. D. (2021). Sociodemographic determinants of dietary diversity and meal frequency among mothers/caregivers paired with infants and child age 6 to 23 months. *International journal of nutrition and food sciences*, 10(1):1.

[52] Woldegebriel A. G., Desta A. A., Gebreegziabiher G., Berhe A. A., Ajemu K. F. and Woldearegay T. W. (2020). Dietary diversity and associated factors among children aged 6–59 months in Ethiopia: analysis of Ethiopian demographic and health survey 2016 (EDHS 2016). *International journal of pediatrics*, Article ID 3040845. doi: 10.1155/2020/3040845 32908551 PMC7474757

[53] Zebadia E., Mahmudiono T., Atmaka D. R., Dewi M., Helmyati S. and Yuniar C. (2021). Factors associated with minimum acceptable diet in 6–11 months old Indonesian children using the 2017 IDHS. *Open access Macedonian journal of medical sciences*, 9(E):1403–12.

[54] WHO (2017). Global Nutrition Monitoring Framework: Operational Guidance for Tracking Progress in Meeting Targets for 2025. World Health Organization, Geneva, Switzerland.

